# Further characterization of changes in axial strain elastograms due to the presence of slippery tumor boundaries

**DOI:** 10.1117/1.JMI.5.2.021211

**Published:** 2018-02-05

**Authors:** Christopher Uff, Leo Garcia, Jeremie Fromageau, Aabir Chakraborty, Neil Dorward, Jeffrey Bamber

**Affiliations:** a Institute of Cancer Research and Royal Marsden NHS Foundation Trust, Joint Department of Physics, Sutton, Surrey, United Kingdom; b Southampton General Hospital, Department of Neurosurgery, Southampton, United Kingdom; c National Hospital for Neurology and Neurosurgery, Victor Horsley Department of Neurosurgery, Queen Square, London, United Kingdom

**Keywords:** ultrasound elastography, contrast transfer efficiency, mobile boundary, slip plane

## Abstract

Elastography measures tissue strain, which can be interpreted under certain simplifying assumptions to be representative of the underlying stiffness distribution. This is useful in cancer diagnosis where tumors tend to have a different stiffness to healthy tissue and has also shown potential to provide indication of the degree of bonding at tumor–tissue boundaries, which is clinically useful because of its dependence on tumor pathology. We consider the changes in axial strain for the case of a symmetrical model undergoing uniaxial compression, studied by characterizing changes in tumor contrast transfer efficiency (CTE), inclusion to background strain contrast and strain contrast generated by slip motion, as a function of Young’s modulus contrast and applied strain. We present results from a finite element simulation and an evaluation of these results using tissue-mimicking phantoms. The simulation results show that a discontinuity in displacement data at the tumor boundary, caused by the surrounding tissue slipping past the tumor, creates a halo of “pseudostrain” across the tumor boundary. Mobile tumors also appear stiffer on elastograms than adhered tumors, to the extent that tumors that have the same Young’s modulus as the background may in fact be visible as low-strain regions, or those that are softer than the background may appear to be stiffer than the background. Tumor mobility also causes characteristic strain heterogeneity within the tumor, which exhibits low strain close to the slippery boundary and increasing strain toward the center of the tumor. These results were reproduced in phantom experiments. In addition, phantom experiments demonstrated that when fluid lubrication is present at the boundary, these effects become applied strain-dependent as well as modulus-dependent, in a systematic and characteristic manner. The knowledge generated by this study is expected to aid interpretation of clinical strain elastograms by helping to avoid misinterpretation as well as provide additional diagnostic criteria stated in the paper and stimulate further research into the application of elastography to tumor mobility assessment.

## 1 Introduction

Quasi-static ultrasound elastography generates images of tissue strain, referred to as elastograms, which aim to reflect the underlying relative tissue stiffness.^1^ This is achieved by gently palpating the tissue with the ultrasound transducer and simultaneously monitoring tissue displacements from the received echo data. Typically, the absolute axial component of normal strain is displayed, where axial is defined as both the direction of beam propagation and applied force.^1^^–^^5^

Strain is a useful quantity to measure due to its dependence upon tissue stiffness, which is clinically relevant because pathological changes are often accompanied by altered tissue stiffness.^6^^,^^7^ The term elastography has since been used to describe a wide range of biomechanical imaging methods including deep loading of the tissue using acoustic radiation force^8^^,^^9^ and those that display: shear elastic modulus by measuring the speed of shear waves,^10^^–^^12^ Young’s modulus (YM) reconstructed from induced tissue displacements,^13^^,^^14^ the nonlinearity of the stress–strain relationship,^5^ Poisson’s ratio,^15^ shear strain,^16^^–^^19^ shear viscosity,^20^ and tissue permeability to mobile fluid.^21^^–^^28^ A summary and comparison of the various methods may be found in Ref. 29. Ultrasound elastography, whether imaging tissue elasticity quantitatively or as relative stiffness or strain, is proving to be important in a variety of clinical applications, such as improving tumor detection and characterization,^7^^,^^30^^–^^34^ and detecting and assessing cardiovascular disease.^16^^,^^35^

The degree of bonding of a tumor to the background tissue, also described as the degree of tumor mobility, where a tumor is regarded as mobile if it is mechanically poorly integrated with (i.e., not bonded to) the surrounding tissue, has been used to help differentiate between benign and malignant tumors;^36^ local infiltration of malignant tumors, for example, is believed to increase mechanical integration between tumor and surrounding tissue. To evaluate the degree of bonding between different tissues is therefore potentially useful for differential diagnosis of breast tumors, for example, because benign tumors tend to be more mobile than malignant tumors.^36^^–^^39^ It may also have application in surgical guidance, where knowledge of potential planes of cleavage is extremely useful.^40^^,^^41^ In a finite element model (FEM) study, the degree of bonding of an inclusion to its surroundings significantly affected the axial normal strain image contrast between tumor and background,^18^ and quasistatic elastography has generally shown potential to provide tumor mobility information.^17^^,^^42^^–^^44^ A number of elastographic features have now been identified as being associated with the degree of bonding between tumor and the surrounding tissue, as follows.

Malignant tumors often appear larger on elastograms than the corresponding sonograms.^6^^,^^45^ The explanation usually given is the spread of spicules from the tumor or region of desmoplasia at the margin, which are hard to detect on B-mode images. Benign tumors tend to appear the same size in the two types of image. This difference in apparent tumor size has been suggested to represent new information provided by elastography, which improves ability to noninvasively distinguish between benign and malignant breast lesions. In fact, the role of elastography in this respect may well be that of drawing attention to, or improving visual perception of, ultrasound image information that was already employed for diagnosis in some centers long before elastography was developed; it has been known since the beginnings of grayscale breast ultrasound that the true size of a tumor is often not its hypoechoic nidus but a larger region where the nidus is sometimes bounded by a rim or halo that is hyperechoic relative to the breast tissue, and where the largest discrepancies between the two size-measures often occur in the lateral direction.^46^^,^^47^ The detection of a hyperechoic rim is one of the most predictive echographic features of malignancy,^48^ but this feature is more difficult to identify (e.g., in the dense breast) when the tumor is surrounded by hyperechoic parenchyma. In this case, however, observation on real-time, rather than static, echography may allow this rim or halo to be identified by the way that it moves during palpation.^38^^,^^49^ In other words, this may be the same information as that provided by elastography, although elastography may display it in a manner that is easier to perceive, just as elastography improves the perception of relative stiffness over that achieved with real-time B-mode relative motion assessment.^50^

**Fig. 1:**
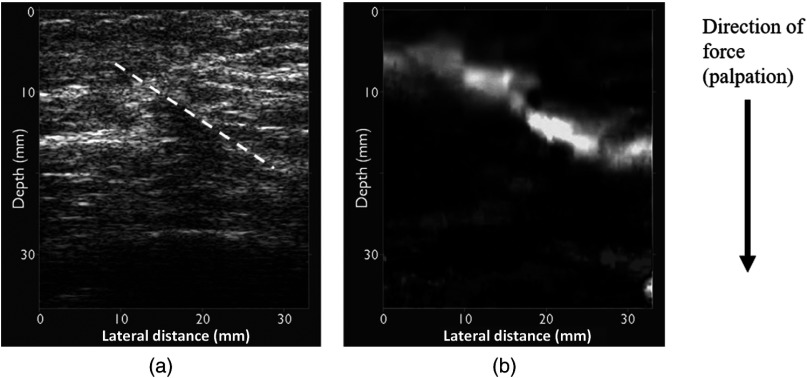
Postoperative breast scar. (a) B-mode echogram. During palpation the real-time image demonstrated a visually obvious diagonal line of separation of direction of speckle motion (speckle shear), which passed through an echogenic and acoustically shadowing region believed to be scar tissue. When applying compressive strain to the tissue using the ultrasound probe, tissue to the right of, and above, this line (indicated here by the dashed white line) appeared to be sliding over (i.e., to the right of, and down, with respect to) tissue to the left of, and below, this line. (b) The corresponding freehand elastogram, calculated as the absolute value of axial strain, scaled between 0% (black) and 0.88% (white). Other details of the system and software used to generate the elastogram may be found in Ref. 52. A diagonal line of high axial strain may be seen to coincide with the line about which speckle shear was observed to have occurred in the real-time B-scan. The elastogram also demonstrates little strain elsewhere in the image, suggesting that slip occurred in preference to strain. These appearances are similar to those observed for strain images of gelatine phantoms that have been cut diagonally and subject to compressive stress sufficient to cause slip between the two parts of the phantom.^53^ (Images courtesy of Bamber et al.^52^)

It has also been observed in a preliminary simulation study that whether or not an inclusion is attached to its surroundings significantly affects the axial strain image contrast between the inclusion and the background^16^^,^^44^ and also found that mobile inclusions showed a “mechanical artifact” of high axial strain at the boundary, which was attributed to “shear strain.” This artifact was also demonstrated in phantom images (Fig. 9 in Ref. 51) and an image of a postoperative scar in breast tissue shown in Ref. 52 reproduced in Fig. 1, where it was also attributed to tissue shear. The artifact was absent in the results of a simulation study of the effect of inclusion boundary conditions on axial strain images,^18^ although in that study it was shown that axial strain is heterogeneously distributed inside a mobile inclusion and that estimating modulus contrast from axial strain images of mobile inclusions is influenced by the location of the region of interest (ROI) chosen to represent the background tissue.

Taking the lateral gradient of axial displacement data yields a quantity called axial shear strain. This parameter has also shown potential to provide mobility information due to differences observed between axial shear strain elastograms from mobile and adhered inclusions^16^^,^^17^ and certain image features, such as the total area of positive and negative axial shear strain, may be extracted as parameters to aid discrimination between adhered and mobile tumors. Furthermore, axial shear strain “fill-in” within the interior of tumor has been observed when the tumor is elliptical, mobile, and at a nonnormal orientation,^54^ which may also provide a method of determining the adherence of a tumor.

Currently, absolute axial normal strain is displayed by all commercially available ultrasound quasistatic elastography systems. Further research into the impact of lesion mobility on this quantity may therefore enlighten and assist those using elastography. Diagnostic performance may be improved through additional knowledge of how to extract mechanical tissue parameters from conventional elastography. Our overall (eventual) aim is therefore to investigate whether, and how, elastography can be used to detect a mobile tumor’s slippery boundary and characterize regions of slip in terms of their coefficient of friction. In this paper, we use FEM and a tissue-mimicking gelatine phantom that simulates a similar model to the FEM, to extend current understanding of how axial strain images are affected by the presence of slippery boundaries, and how such strain images vary with applied strain and Young’s modulus contrast. To quantify those changes, the contrast-transfer-efficiency (CTE) curves, which are defined below, of a mobile and adhered inclusion are compared. To our knowledge, this comparison has not been made before. Strain contrast between the slip-induced strain halo and the background, as a function of Young’s modulus contrast and magnitude of applied strain, is also examined, and the mechanism of origin of this strain halo is elucidated. We examine the similarities and differences between FEM and experimental data with reference to the conditions that result in these similarities and differences.

The FEM in this study is limited to two-dimensional (2-D) plane strain simulations, even though any incompressible medium will undergo three-dimensional (3-D) deformation when a unidirectional stress is applied to it. We regard this as valid since, for the phenomena studied, the trends in the results should generalize to 3-D; 2-D plane strain models have been similarly used to predict many aspects of the full 3-D mechanical behaviur.^17^^,^^55^ Since we wish to establish a “ground-truth” to the elastographic appearance of slippery boundaries in the absence of noise, we have chosen to obtain results by analyzing noiseless FEM-generated displacement data, without simulating the processes of ultrasound image formation and estimation of displacement and strain. The experimental data to which these results are compared are necessarily generated from analysis of ultrasound images.

## 2 Materials and Methods

### 2.1 Finite Element Model Simulation

Comsol Multiphysics v3.5a (COMSOL) was used to generate a 2-D plane strain model (see Appendix A), consisting of a circular inclusion (1.5 cm radius) embedded at the center of a square homogeneous background (10 cm). The simulation comprised a 2-D triangular element mesh using Comsol’s extremely fine predefined element size. Both the inclusion and background were linear, isotropic, and quasi-incompressible, with their Poisson’s ratio, ν, set to 0.495 The Young’s modulus of the background was 10 kPa and varied from 1 to 100 kPa in the inclusion between successive simulations. Two versions of this model were simulated: perfect inclusion-background adhesion and perfect inclusion-background slip (that is, the inclusion boundary could slip against its surroundings with a coefficient of friction of zero).

Simulations featuring discontinuous media must be performed using contact modeling to simulate the motion of two bodies sliding past each other. Contact modeling entails mesh discontinuity across the contact boundary, in other words, elements do not exist across the boundary between contact bodies. As such, care must be taken with regards to interpretation of measurements from strain maps that are formed directly from mesh deformation data compared to those from strain images generated through spatial gradient analysis of displacement data. The latter is generally the method employed in elastography, and attention must be given to the fundamental difference in strain calculation where contact bodies are present.

The model was exposed to a uniaxial compressive force across the entire upper surface and the resulting axial displacement and strains predicted throughout the model. Global applied strain values of 0.1%, 0.2%, 0.5%, 0.8%, and 1% were simulated. Slippery boundary conditions were assigned to the upper and lower model surfaces, and rigid body motion was prevented by laterally confining a node at the center of the lower boundary and a node in the center of the inclusion. Axial strain was calculated in two ways: the “element-based” method of the FEM package and through exporting the nodal displacement values to MATLAB^®^ (Mathworks, Massachusetts) for conversion to strain via a least-squares estimator with a window length of 5 mm, which we call “gradient-based” strain. Although it has not previously been employed in FEM simulations of strain images in the absence of simulation of ultrasound echoes, the latter method was used because most elastography systems estimate strain in this way and, without it, the high strain halo seen in practice and mentioned above is not created.

The effect of slip-induced strain at the tumor boundary on CTE values was examined through calculating CTE using both element-based strain images directly from FEA, and gradient-based strain images. CTE is defined as the ratio (in decibels) of the mean lesion to background strain contrast to the equivalent lesion to background Young’s modulus contrast^56^

$$\mathrm{CTE}\;(\mathrm{dB})=|C_{\epsilon }\;(\mathrm{dB})|-|C_{\mathrm{YM}}\;(\mathrm{dB})|,$$ (1)

where

$$C_{\epsilon }=\epsilon_{\text{tumor}}/\epsilon_{\text{background}},\;C_{\mathrm{YM}}=E_{\text{tumor}}/E_{\text{background}},$$

ϵ is strain, $C_{\epsilon }$ is the strain (image) contrast, E is the value of Young’s modulus. and $C_{\mathrm{YM}}$ is the Young’s modulus (object) contrast.

The elastographically observed strain contrast, $C_{\epsilon }$, is the ratio of strain observed in the background that is observed to be transmitted into the tumor. The true YM contrast, $C_{\mathrm{YM}}$, is the ground truth and must be known. The fundamental concept of CTE is to define the fidelity with which axial strain images (elastograms) reproduce the true mechanical contrast present in the material, expressed as a ratio. Although it is impossible for CTE to be measured clinically because the ground truth ($C_{\mathrm{YM}}$) cannot be known, it is an important property that determines the reliability of axial strain images under certain conditions, many of which arise clinically.

For studying the strain halo, mean strain values within an annulus centered on the inclusion boundary, of width equal to half the strain estimator length, were calculated. For studying CTE, ROIs were defined as a disc within the inclusion, avoiding the inclusion boundary, for measuring $\epsilon_{\text{tumor}}$ and an annulus surrounding the inclusion, again avoiding the boundary, for measuring an average $\epsilon_{\text{background}}$. The variation in these quantities with the $\epsilon_{\text{tumor}}$ ROI radius was also studied, given the expected heterogeneity of strain distribution and the resulting effect upon calculated strain values. Throughout the remainder of this paper, “strain” refers to absolute axial normal strain.

### 2.2 Tissue-Mimicking Gelatine Phantoms

In order to generate comparable data to the FEM simulations, prismatic gelatine phantoms were constructed in which the YM varied in the cross section that formed the ultrasound scan plane but was constant in the elevational direction. This provided consistency with the plane strain assumption used to predict the mechanical behavior in the FEM simulations. These phantoms comprised a cube containing a centrally placed circular rod-shaped inclusion, and the YM of the rod and cube were varied to generate phantoms of varying YM contrast. The use of a rod-shaped inclusion rather than a spherical inclusion was decided on for several reasons. First, this mimics the FEM simulation, second the conclusions drawn from results from the rod-shaped inclusion are on the whole generalizable to spherical inclusions and in addition to this there are numerous clinical situations where the ROI is more similar to a rod than to a sphere (for example, tendons). It is not thought that the difference will significantly affect the trends seen for CTE.

### 2.3 Young’s Modulus Measurement

In order to establish a gold truth, measurements of the YM of the gelatine were made using gelatine cylinders manufactured from the same batch of gelatine that the phantoms were constructed from. Following the method described by Crescenti et al.,^57^ cylinders of gelatine 35 mm in height and 53.5 mm in diameter were made at the concentrations of 8%, 10%, 12%, and 14%. YM was measured from force-displacement data obtained by controlled application of compressive strain along the long axis of the cylinders, and from force measurement (Instron 3342 with load cell 2519-103, Instron, Bucks, United Kingdom). 3% prestrain was used to ensure uniform contact between the compression plate and the sample. Both the compression plate and the base plate were larger than the surface of the gelatine, and they were lubricated liberally with vegetable oil to avoid measurement artifacts due to boundary friction, as described by Crescenti et al.^57^ The force (measured by the load cell) and the displacement were recorded every 20 ms during a 5% strain. Thus, including prestrain, a total of 8% strain was applied. Each sample was strained six times for repeatability evaluation and between each test the sample was removed, repositioned, and relubricated. The YM values were selected to represent a similar range to those found in biological tissues.

### 2.4 Phantom Construction

In order to make the phantoms, the background mixture was first poured into cuboidal moulds of side 60 mm. A 15-mm-diameter rod was suspended in the liquid so when it was removed, a well was left in the cube. Adhered inclusions were made by pouring liquid gelatine into the well at 33°C and allowing it to set at room temperature. Mobile inclusions were made by pouring liquid gelatine into the well at 28°C (the point when it was about to set). Once set, the phantom was immersed in a water bath, and a slip plane was developed between the inclusion and the background by manually releasing the inclusion with gentle force applied along its axis using a 2-mm-diameter metal rod. This did not constitute cutting the gelatine as gentle manipulation, as well as the presence of water allowed the layers to separate into inclusion and background with no damage to either layer. This was evidenced by the absence of any split in the gelatine on B-mode ultrasound imaging. Once the inclusion had been released, it was possible to see it moving relative to the background. The inclusion was released at the last possible moment in order to minimize the possibility that significant amounts of the gelatine would dissolve into the water.

A set of seven adhered and mobile inclusion phantoms with different contrast were made, detailed in Table 1. The Perspex moulds in which the phantoms were made were refrigerated in airtight containers at 4°C prior to experiments and were removed from the refrigerator at least 4 h prior to scanning to allow them to reach room temperature. They were removed from the Perspex moulds and the internal temperature was monitored to be the same as the ambient temperature of 20°C by inserting the needle thermocouple of a digital thermometer into the phantom at a point not included in the scan plane to avoid artifact from this.

**Table 1:** Young’s modulus contrast ($C_{\mathrm{YM}}$) in the phantoms.

% gel (background)	YM (kPa)	% gel (inclusion)	YM (kPa)	$C_{\mathrm{YM}}$ (dB)
8	22.8	14	61.1	4.27
8	22.8	12	47.6	2.68
8	22.8	10	32.9	1.08
14	61.1	14	61.1	0
14	61.1	12	47.6	−1.58
14	61.1	10	32.9	−3.18
14	61.1	8	22.8	−4.27

### 2.5 Image Acquisition

Each experiment was performed with the phantoms resting on an extremely stiff acoustic absorbent pad while immersed in a water bath at constant temperature of 20°C. Frames of radio frequency (RF) data were acquired using a Gage Compuscope 14200 (Cage Applied, Lockport, Illinois) in a personal computer running Stradwin 3.8 (Cambridge University, United Kingdom) ultrasound elastography and image acquisition software,^58^ interfaced to a DIASUS (Dynamic Imaging, United Kingdom) scanner. A GE RSP6-12 mechanically swept 3-D probe (GE Healthcare, Chalfont St Giles, United Kingdom) was used in 2-D mode with the internal linear array (6 to 12 MHz) held stationary in the central position; in effect a linear array transducer with a footprint extender that covered the upper surface of the phantom. The transducer was attached to an Instron In-Spec 2200 Benchtop Portable Tester (Instron, Bucks, United Kingdom). One Newton of precompression was first applied to establish acoustic and uniform mechanical contact between the transducer and the phantom, which was comparable to the 3% prestrain during YM measurement. An image frame of RF ultrasound echo data was acquired at this position and this image was then considered as the reference frame, i.e., 0% deformation, for strain measurement in RF images acquired at the end of each of 10 subsequent compression steps of 0.5% (0.3 mm). All RF frames were sampled in the axial direction at 66.6 MHz and comprised 127 A-lines, corresponding to an image of 2.5 cm width and 6.5 cm depth. The applied force at each compression step was recorded.

### 2.6 Image Analysis

Axial displacements were estimated between consecutive frames of RF data using an exhaustive search 2-D crosscorrelation echo-pattern tracking algorithm written in MATLAB^®^ (Mathworks, Massachusetts). Further details of the algorithm may be found in Ref. 3. The tracking parameters were search window 250×10, reference window 120×5, step axial 5, and step lateral 2. Axial normal strain images were then derived from the displacement data using a moving least-squares strain estimator with a window length of 50.

Each strain image frame was then read into ImageJ 1.44 (bundled with 32-bit Java 1.6.0_20) image analysis software at a resolution of 250×650  pixels (10 pixels per mm). Two ROIs were defined for calculating the mean strain within the inclusion and background. A circular ROI, whose diameter was varied to investigate different regions within the inclusion, was used for the inclusion ROI. An annulus-shaped ROI of 20 pixels width (equal to approximately half the strain estimator window length), centered on the inclusion boundary, was used to measure strain across the inclusion boundary, and an annulus of width 50 pixels, starting at the outer boundary of that ROI was used to measure background strain. It was found that using ROIs from a single region of the background produced results that were not representative of the whole background and caused strain measurements to vary considerably. The approach of using circles and annuli resolved such problems for the phantom employed, which produced strain images that exhibited circular, axial, and lateral symmetry.

CTE was calculated using the method described by Kallel et al.^59^ Mean strain values within the annulus centered on the inclusion boundary were used as a surrogate for mobility and were first plotted as a function of applied strain for each value of $C_{\mathrm{YM}}$ and then, after these results were used to select appropriate frames and regions for further analysis, as described below, CTE was plotted as a function of $C_{\mathrm{YM}}$. In this way, CTE results were compared for soft and hard inclusions, both adhered and mobile.

## 3 Frame and Region of Interest Selection

Although the applied interframe strain magnitude did not vary, some compression-dependent behavior was observed, as described below. As such, frame and ROI selection criteria were developed to guide the analysis of the results. In adhered inclusion phantoms, there was little applied strain-dependent behavior, whereas in mobile inclusion phantoms, strain distributions tended to change with applied strain, the most significant change being loss of the slip plane at high strains and applied forces, sometimes for the whole boundary displayed in a given frame and sometimes for only a portion of the boundary. Hence, the presence of a high contrast strain halo across the inclusion boundary, which from the FEM results corresponds to a slip plane was deemed to signify an inclusion that was mobile either as a whole or in part. We further discuss the rationale for our frame and region selection in the results section.

## 4 Results

### 4.1 Finite Element Model Data

**Fig. 2:**
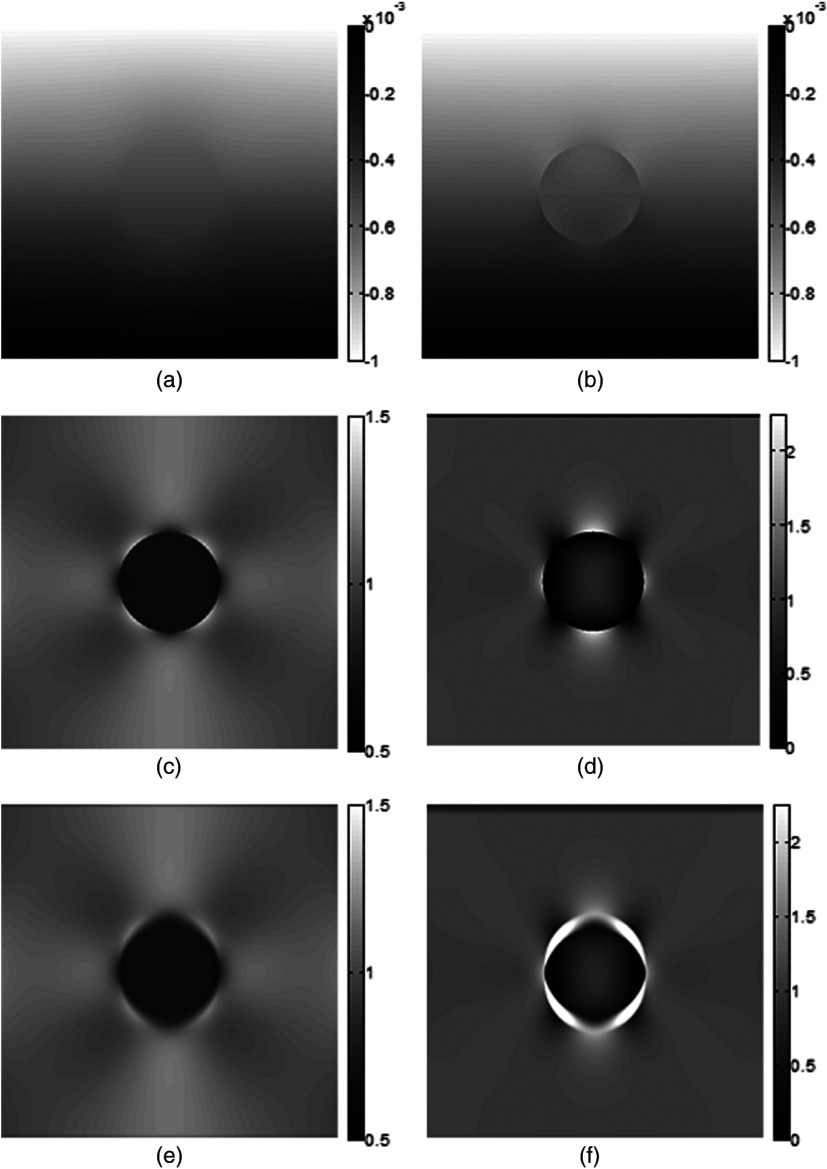
Left column: adhered model, right column: slippery model. (a) and (b) Axial displacement; (c) and (d) axial strain as calculated by element-based strain estimator and (e) and (f) axial strain as estimated by gradient-based strain estimator. Note differences in scale between adhered and slippery models. Scale in %.

In Fig. 2, axial displacements and strains from a model with inclusion modulus 20 kPa and 1% applied strain (induced by a 1-mm displacement of the top surface of the model in a downward direction) are displayed, and results from element-based and gradient-based strain estimation can be compared. The mobile inclusion demonstrates a discontinuity in displacement data along its boundary [Fig. 2(b)], which is not present for the adhered inclusion [Fig. 2(a)] where there is a smooth gradient in displacement across the boundary. The discontinuity observed in the axial displacement field causes a halo of high axial strain to surround the mobile inclusion in the gradient-based strain image [Fig. 2(f)], which is not seen in the element-based strain image [Fig. 2(d)].

**Fig. 3:**
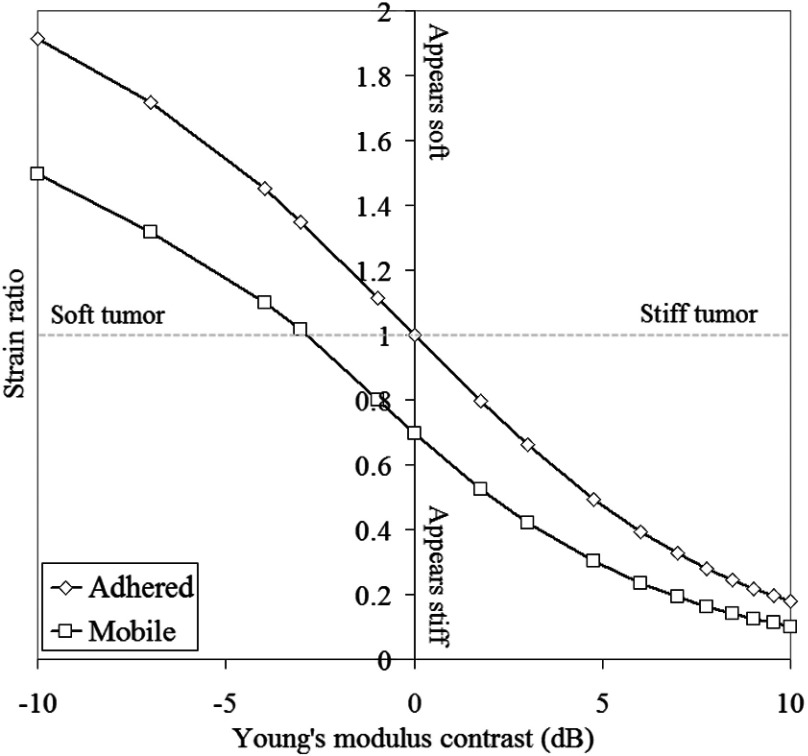
Mean absolute inclusion to background axial strain ratio (i.e., contrast) as a function of Young’s modulus contrast, due to a 1% applied compression, for adhered and mobile models. The terms stiff, soft, appears stiff and appears soft, apply to the inclusion and are relative to the background. Data taken from FEM “element-based” strain.

Furthermore, there are differences in axial strain distribution between the mobile and adhered inclusion models. Inside the inclusion, for the adhered case [Fig. 2(c)], strain is homogeneously distributed, whereas in the mobile case [Fig. 2(d)] strain reaches a maximum in the inclusion center, even though the Young’s modulus is constant throughout. In the background tissue, it can be seen that the pattern of the stress concentrations also changes: it is homogeneous over more of the background in the mobile case [Fig. 2(d)] than in the adhered case [Fig. 2(c)]. In the mobile case, the strain variations, although more pronounced than in the adhered case, are located mainly in the areas close to the inclusion and decrease rapidly away from the inclusion boundary. On the circular edge of the inclusion, the locations of high and low strain regions are inverted compared to the adhered case. This inversion in the locality of the boundary is seen clearly for element-based strain [Figs. 3(c) and 3(d)], but Fig. 2(f) suggests that, depending on the resolution limit imposed by the strain estimator window size, the feature may be partially or completely obscured by the bright strain halo in the experimental elastograms. It is also interesting to note the similarity of the pattern shown in Fig. 2(f) to that seen in experimental elastograms of slippery boundaries (see Fig. 1), indicating that mechanisms such as that suggested by Konofagou for explaining the strain halo, although plausible, are not necessary, i.e., the halo may simply be a consequence of the interaction between the gradient-based strain estimator and the displacement discontinuity at the slippery boundary, seen in Fig. 2(b). Finally, for the mobile case, even far from the boundary, there is a partial inversion of the locations of high and low strain regions in the background, as compared to the adhered case. This is best seen in Fig. 2(f), as compared with Fig. 2(e); note that in the upper right quadrant of Fig. 2(e), beginning with the vertical direction as 0 deg, one can see bright (0 deg), dark (45 deg), and bright (90 deg), whereas in Fig. 2(f) the angular variation is bright (0 deg), dark (30 deg), bright (45 deg), and dark (90 deg). This pattern for the mobile inclusion is repeated in other quadrants and is also visible in the experimental elastograms of mobile inclusions (see Figs. 8, 9, and 11). However, when viewing axial strain images using the same scale, the differences in the tissue background strain distribution are less obvious, although the strain distribution differences in the inclusion are still easily visible.

Figure 3 shows mean inclusion to background strain ratios calculated directly from FEM data, where strain in the inclusion was averaged from all mesh elements located in the inclusion, and similarly strain in the background was averaged from all elements located in the background. The applied strain was fixed at 1%. At all values of YM contrast, there is a reduction in mean strain ratio as a consequence of tumor mobility, despite the visible high strain concentration toward the center of the mobile tumor [Fig. 2(d)]. It can also be seen that, where the inclusion and background have identical Young’s moduli, a nonzero strain ratio is calculated for the mobile case. Recall that this result is generated in the absence of any strain generated in the image as a consequence of passing the gradient window of the strain estimator over the tumor boundary in the displacement data.

**Fig. 4:**
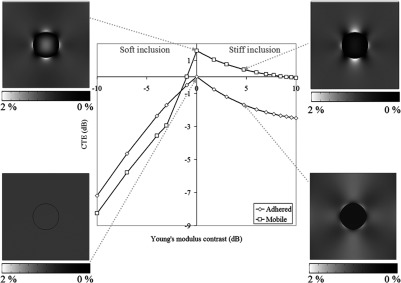
Contrast transfer efficiency curves for adhered and slippery models. Applied strain for both models is 1%. (Element-based) strain images corresponding to points of interest, as indicated.

The CTE curves for the adhered and slippery models are compared in Fig. 4. The CTE of the adhered model is always negative, whereas in the mobile case the CTE is positive for modulus contrasts>−0.97  dB. It is shown that, for a Young’s modulus contrast of 0 dB (i.e., the inclusion is the same stiffness as the background), the slippery model has a CTE of 1.58 dB. The adhered and slippery models have the same CTE values for a modulus contrast of −1.49  dB, although it must be noted that this does not mean that the images are identical, only that the calculated CTE values are identical. The slippery inclusion has a CTE of approximately zero for Young’s modulus contrast values of −0.97 and 9.03 dB, although this does not mean that the strain images appear homogeneous, either in the background or within the inclusion (as is the case for the adhered model at Young’s modulus contrast=0  dB, where the strain image really is homogeneous and CTE=0  dB). The point where the two CTE curves cross and where the mobile model curve crosses 0 dB CTE may change with variables such as inclusion size, position, etc., and does not mean that the two images are identical.

**Fig. 5:**
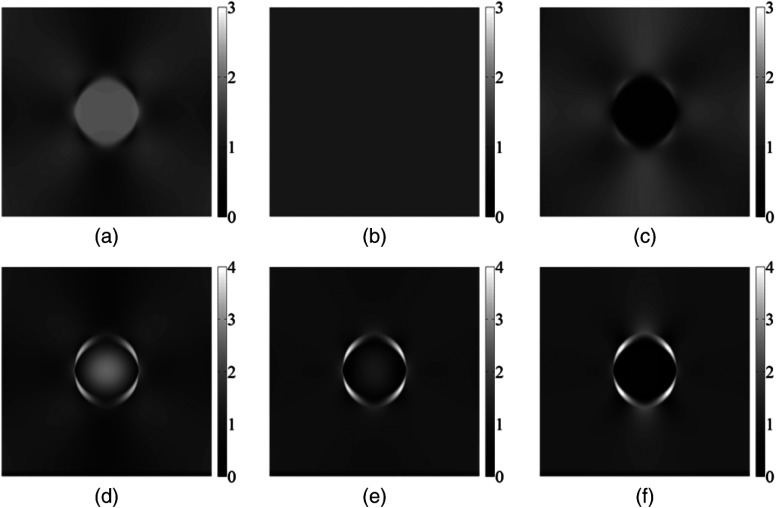
Strain images obtained using gradient-of-displacement for inclusions undergoing 1% strain: (a) background 10 kPa, inclusion 1 kPa, adhered boundary, (b) background 10 kPa, inclusion 10 kPa, adhered boundary, (c) background 10 kPa, inclusion 100 kPa, adhered boundary. (d) background 10 kPa, inclusion 1 kPa, mobile boundary, (e) background 10 kPa, inclusion 10 kPa, mobile boundary, and (f) background 10 kPa, inclusion 100 kPa, mobile boundary.

**Fig. 6:**
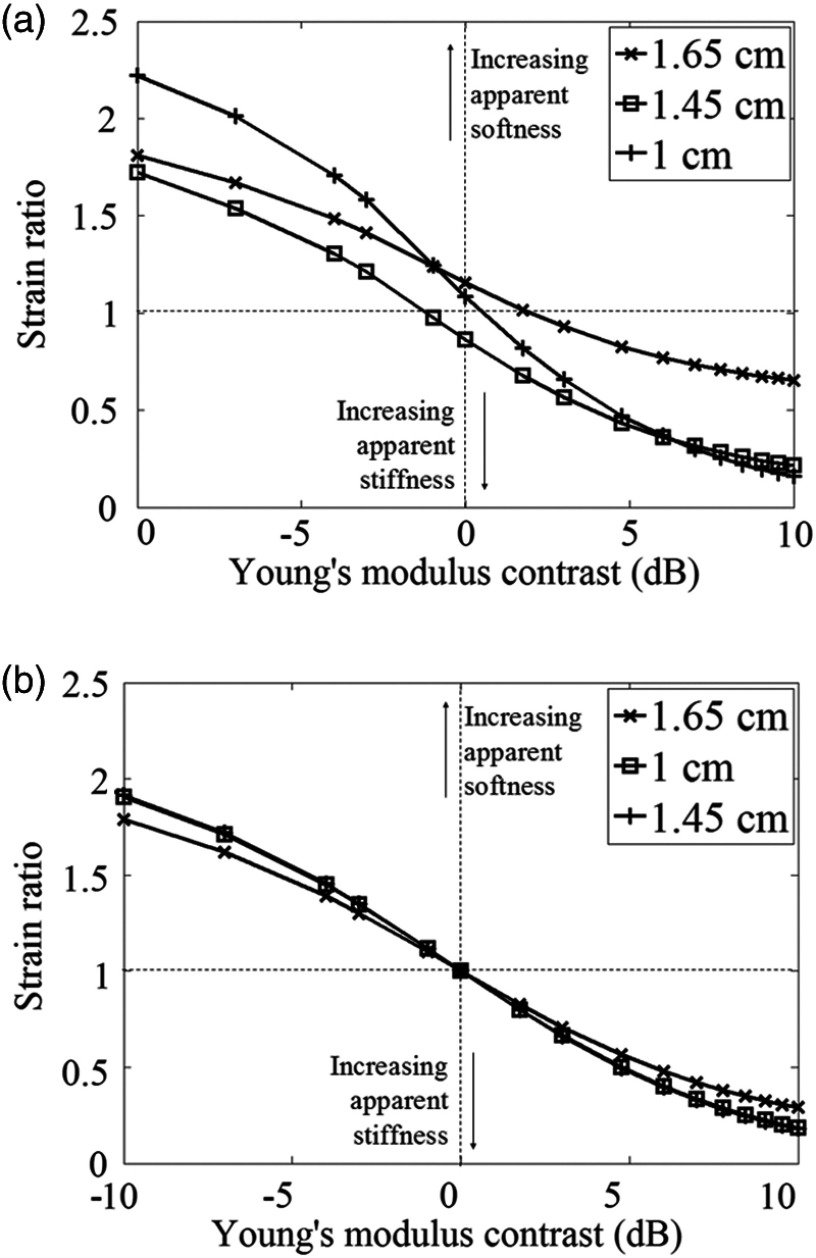
Strain ratio measurements made with a constant background ROI and varying inclusion ROI radii, for (a) mobile and (b) adhered models. Strain was computed as the axial gradient of axial displacement. The applied strain was 1%.

Figure 5 shows strain images from a 1 kPa [Figs. 5(a) and 6(d)], 10 kPa [Figs. 5(b) and 6(e)], and 100 kPa [Figs. 5(c) and 6(f)] mobile and adhered inclusions, obtained by the gradient-based method. The strain halo is present in all strain images of the mobile inclusion. The characteristic heterogeneous strain distribution within the mobile inclusion is visible in Figs. 5(d) and 6(e). Although it is present in Fig. 5(f), it is not apparent without rescaling the image because the inclusion is very stiff compared to the background. Observe also that the strain magnitude within the slip-induced halo appears approximately independent of the Young’s modulus contrast in the image.

Examining the effect of inclusion ROI size upon strain ratio measurements, Fig. 6 shows the variation in (gradient-based) strain ratio for inclusion ROI radii of 1.65 cm, which completely overlaps the tumor boundary, 1.45 cm, which encompasses the inclusion up to the start of the strain halo, and 1 cm, which is within the center of the inclusion, for mobile and adhered models. The background ROI was kept constant at the area beyond a 1.75-cm radius from the model center, which ensured that the strain halo was not included in any background strain measurement. For the mobile case, when the inclusion ROI encompasses the strain halo, the strain ratio increases, indicating an apparent softening effect, under the assumption that strain ratios are (naively) interpreted as stiffness ratios. When the inclusion ROI is 1 cm, much of the strain within the inclusion is neglected, resulting in values that differ considerably from those in Fig. 2. It is only when the inclusion ROI is set at a value that ensures that the strain halo is neglected entirely while maximizing the total area of the inclusion within it, that strain ratio values similar to that generated from FEM are obtained. In Fig. 6(b), it is seen that the adhered model strain ratio measurements are not as sensitive to inclusion ROI size, which confirms expectations from visual inspection.

**Fig. 7:**
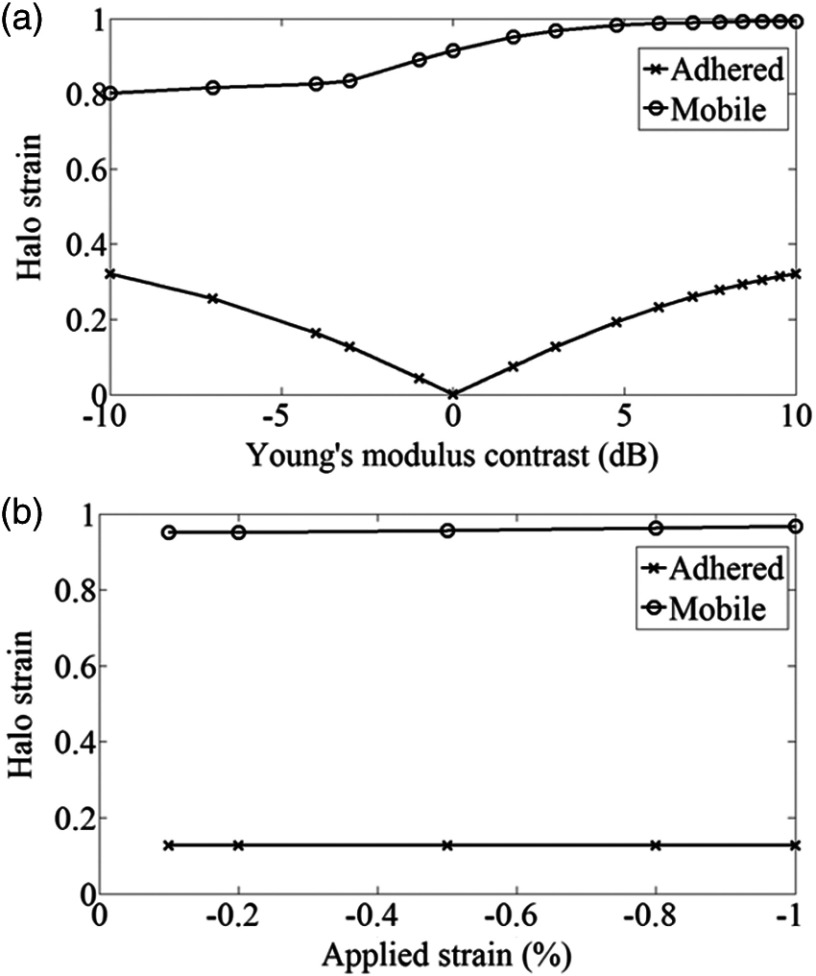
Variation of halo strain with (a) Young’s modulus contrast at 1% applied strain and (b) applied strain for an inclusion of modulus 20 kPa. The modulus of the background was 10 kPa.

**Fig. 8:**
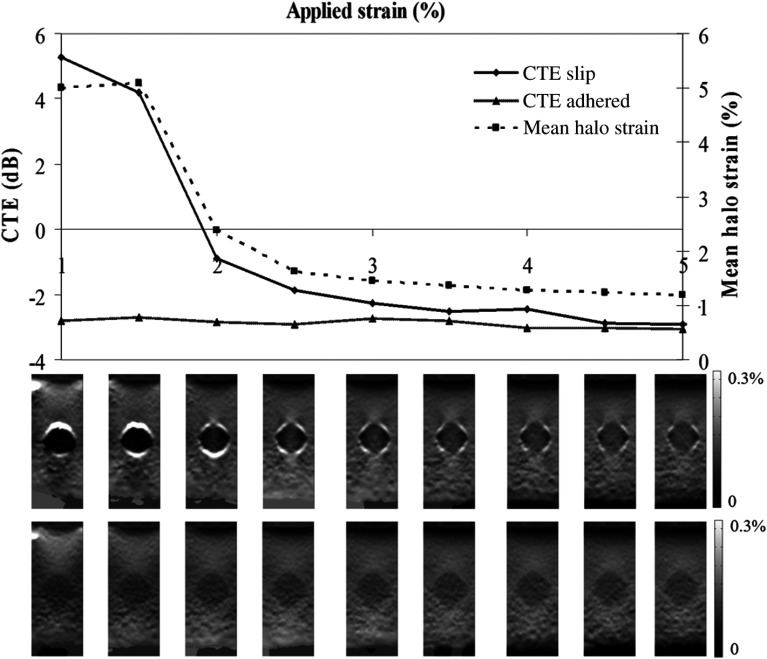
CTE variation with applied strain for phantoms where the inclusion is stiffer than the background: $C_{\mathrm{YM}}=4.27\;\mathrm{dB}$. CTE slip = CTE for the slippery boundary phantom. CTE adhered = CTE for the adhered boundary phantom. Mean halo strain is the mean strain measured within the annulus centered on the inclusion boundary. The nine images analyzed to produce each set of nine data points are shown as thumbnails at the bottom of the figure, for both the adhered and mobile inclusions. Strain is linear between 0% (dark) and 0.3% (light). Unscaled image is 25  mm×65  mm.

Halo strain measurements with varying Young’s modulus contrast and applied strain are shown in Figs. 7(a) and 8(b). This measurement tends to zero at zero Young’s modulus contrast for the adhered case and is always less than 0.4% when the applied strain is 1%, whereas mobile inclusion halo strain is always greater than 0.8% for 1% applied strain and increases slowly with inclusion modulus. For both model types, halo strain remains approximately constant with applied strain, which is true for any modulus contrast (not shown). Finally, mobile inclusion halo strain is always greater than adhered halo strain.

## 5 Experimental Data

The results of the experiments to determine the YM of the gelatine mixtures used to make the phantoms are displayed in Table 1. Our data follow the square law relationship that has previously been described by Eirich^60^ (Appendix B).

**Fig. 9:**
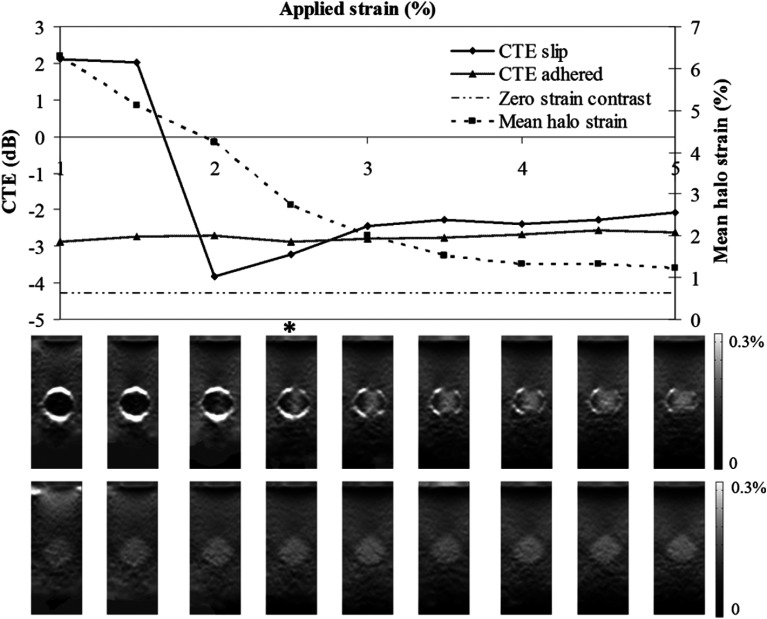
CTE variation with applied strain for a phantom with an inclusion that is softer than the background: $C_{\mathrm{YM}}=-4.27\;\mathrm{dB}$. CTE slip is the CTE for the mobile inclusion phantom. CTE adhered is the CTE for the adhered inclusion phantom. Mean strain halo is the mean strain measured within the annulus centered on the inclusion boundary. Strain is linear between 0% and 0.3%. Unscaled image is 25  mm×65  mm.

Compared with the FEM results where halo strain was seen to be constant with applied strain for both adhered and mobile inclusions, the experimental data agree for adhered inclusions, but mobile inclusions demonstrate considerable variation as a function of applied strain (see Figs. 8 and 9).

In order to understand the differences between FEM and experimental data, the dynamic behavior of the strain in the mobile inclusion phantoms was examined. For both stiff and soft inclusions, the presence of a mobile boundary resulted in reduced strain within the inclusion. If the inclusion is stiffer than the background, a mobile boundary causes the inclusion to appear stiffer than it does with an adhered boundary, i.e., strain contrast increases, causing an increasingly negative $C_{\epsilon }$, as friction at the boundary is reduced. This is illustrated in a reverse sequence from left to right in Fig. 8, where a stiff mobile inclusion ($C_{\mathrm{YM}}=+4.27\;\mathrm{dB}$) has a high CTE initially (i.e., at low strains), and as applied pressure forces the background and inclusion to make contact the mobile boundary is lost with increasing applied axial strain, so CTE rapidly reduces to a level similar to the value seen for adhered boundary inclusions. Note that the initial high CTE values are positive, and yet, CTE is defined by Ponnekanti et al.^61^ as having a maximum value of 0 dB. The interpretation of positive CTE in this context is discussed below. Note also the variation of mean halo strain and the appearance of the halo strain in the thumbnail images, with applied strain; loss of halo strain correlates with loss of $C_{\epsilon }$ and CTE, and this is true even locally, i.e., the inclusion strain increases along the axial lines that pass through regions of decreased halo strain, and the inclusion strain remains low on axial lines that pass through remaining strong halo strain and is illustrated further below where CTE is calculated for these regions separately. Thus, in effect, the phantom shows transitions from a mobile boundary to an adhered boundary, both dynamically and spatially. This behavior is consistent with our FEM data, which suggest that strong halo strain is a good image marker for a mobile tumor boundary. It is used below to guide further analysis of the CTE data.

Similarly, if an inclusion is less stiff than the background (negative $C_{\mathrm{YM}}$), then the presence of a mobile boundary, which causes the inclusion to appear stiffer, acts to reduce the positive contrast. This is seen as reduced inclusion strain in the images, and reduced CTE, from right to left (decreased applied strain), in Fig. 9. Again, regions of strong halo strain appear to indicate slip, since the axial image lines on which they appear also contain regions of reduced inclusion strain, and mean halo strain correlates with reduction in inclusion strain, in this instance resulting in a negative correlation with CTE (because it is a soft inclusion) for applied strains greater than 2%. At applied strains lower than about 2%, in Fig. 9, the correlation between CTE and mean halo strain inverts, becoming positive, and there is an effect on inclusion strain that is so strong that the inclusion (which is the softest of those manufactured), at small applied strains, appears to be stiffer than the background (negative $C_{\epsilon }$), even though it is in reality softer than the background. When a mobile boundary is present, and the conditions are such that applied pressure increases the friction between background and inclusion, there is no consistent relationship between the sign of $C_{\epsilon }$ and the sign of $C_{\mathrm{YM}}$, except at large applied strains where it appears from the halo strain that the mobile boundary is progressively lost with increasing applied strain.

Note also in Fig. 9 that the contrast inversion (negative $C_{\epsilon }$) at low initial strain (0.5% and 1%) leads to high and positive values of CTE. Usually, very high CTE values show that $C_{\epsilon }$ closely approximates $C_{\mathrm{YM}}$. In the present case, however, where contrast is reversed, such interpretation is wrong because these high CTE values are artifactual. Although the inclusion is seen with excellent contrast, this did not arise from the YM contrast. At some point close to 2% applied strain in Fig. 12, the CTE mobile inclusion curve almost touches the zero strain contrast line, at a CTE of −4.27  dB, meaning that the mean strain in the inclusion is the same as the mean strain in the background. For higher values of applied strain, the inclusion correctly appears softer than the background and its CTE curve tends to the adhered case, as it did for the stiff inclusion case of Fig. 8. Meanwhile, in Fig. 9, the CTE for the adhered soft inclusion remains largely independent of applied strain, as it did for the stiff inclusion in Fig. 8, as is expected for gelatine, which is a linearly elastic medium.

Since each compression sequence is based on a single experiment, error bars cannot be given. A measure of the inherent precision of the measurement can be gained from inspecting the CTE values for the adhered phantoms. We therefore believe that the trends seen for the other curves can be trusted.

### 5.1 Contrast Transfer Efficiency as a Function of Young’s Modulus Contrast

**Fig. 10:**
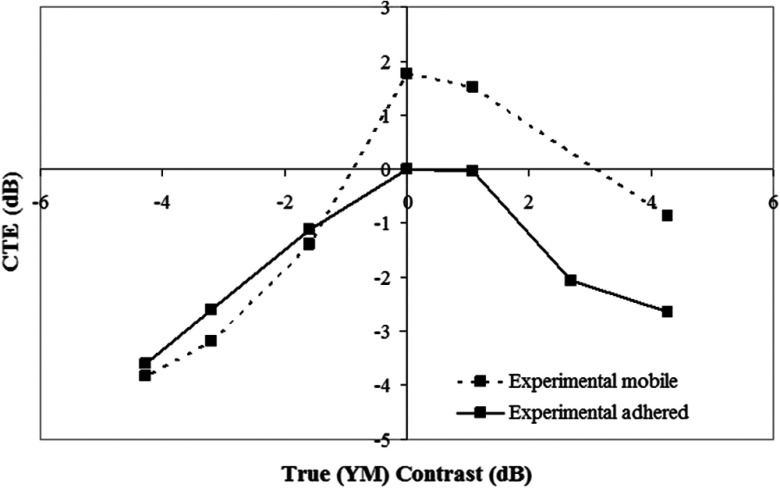
CTE curves for adhered and mobile inclusions with varying Young’s modulus contrast. For mobile inclusions, these data were obtained by measuring CTE from the mean inclusion and mean background strain in the frames selected from dynamic strain sequences where it was judged that excess lubricating fluid had been removed but sufficient remained to ensure a slippery inclusion boundary. For the adhered inclusions, the result was independent of applied strain. For descriptions of how the error bars were calculated, see the main text.

In our FEM simulations of mobile inclusions with a negative $C_{\mathrm{YM}}$, the contrast reversal described above was not observed. We believe that this is due to the presence of lubricating fluid along the mobile inclusion boundary, which was the only way to create low friction in the phantom and was not simulated in FEM. Likewise, for mobile inclusions with positive $C_{\mathrm{YM}}$, the extremely high positive values of CTE at low values of applied strain are believed also to be due to fluid lubrication. As such, for the purposes of plotting CTE as a function of $C_{\mathrm{YM}}$, to make a fair comparison between simulation and experiment, we inspected the elastograms within each dynamic strain sequence for mobile inclusions with negative $C_{\mathrm{YM}}$ and selected for analyzing the frame closest to the contrast reversal point without contrast reversal occurring (i.e., at an applied strain that is equal to or just higher than that at which contrast reversal occurs). This is consistent with the frame in Fig. 9 corresponding to the disappearance of a proximal strain halo (between the transducer and the upper surface of the inclusion) without substantial disappearance of the distal strain halo (deep to the lower surface of the inclusion). This frame is identified in Fig. 9 with “*.” For positive $C_{\mathrm{YM}}$ phantoms, we selected again the frame corresponding to the loss of the proximal strain halo. In Fig. 8, this occurs at an applied strain of about 2%. The rational for these choices was to select frames where excess fluid that we hypothesized was causing the applied strain dependence of the contrast and strain halo (possibly due to its existence as a layer that is “compressible” by virtue of its ability to flow, although this is discussed further below) had been squeezed away but where sufficient lubrication remained such that the inclusions should still be mobile. The CTE values derived from analyzing only these selected frames, for all $C_{\mathrm{YM}}$ phantoms with mobile inclusions, are displayed in Fig. 10. Error bars are not displayed because points are, for the reasons described above, selected from a single experiment involving one set of phantoms. Although each experiment with each phantom had a reliably reproducible trend, the individual points varied such that a direct comparison became under representative of the trend. Also, shown are the CTE values for adhered inclusions, obtained by measuring mean strain in the inclusion and background ROIs over all frames in the sequence. Error bars in this instance were calculated as 1 standard deviation about the mean. They were constantly between 0.10 and 0.14 and are too small to display graphically. There is no point displayed for a mobile boundary phantom with $C_{\mathrm{YM}}=+2.68\;\mathrm{dB}$ because this phantom did not exhibit mobile features. The data from this phantom are very similar to the data from the adhered phantom and so were excluded from the mobile data series.

### 5.2 Stiff Inclusions

**Fig. 11:**
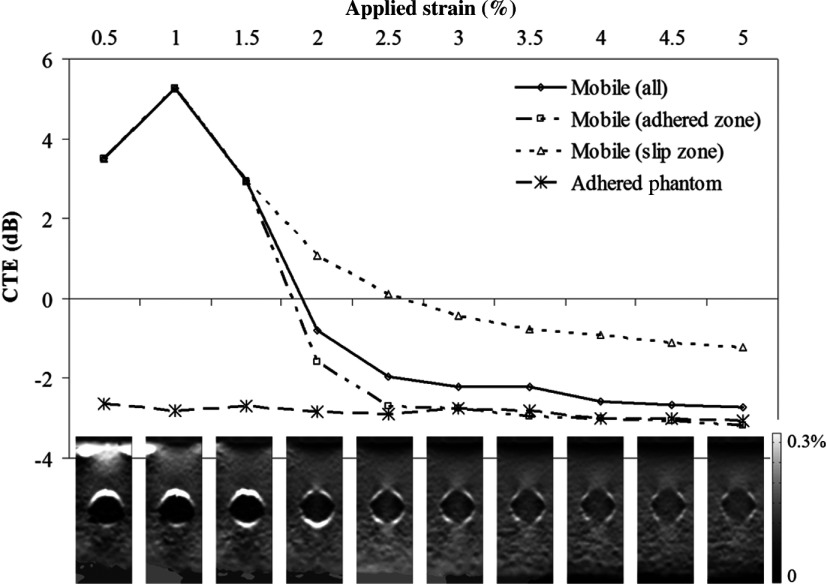
Zones of slip: CTE is plotted against applied axial strain for the slip and adhered phantoms with a YMC of 4.27 dB (stiff inclusion). For the slip phantom, four ROIs were measured: All = the entire inclusion. Slip (adhered zone) = central portion under the zone of adherence. Slip (slip zone) = side regions under the local areas of slip. Slip halo strain = mean strain in the slip halo. CTE for the adhered phantom is also plotted. Strain is linear between 0% and 0.3%. Unscaled image is 25  mm×65  mm.

Figure 11 plots the variation in CTE with applied strain where CTE has been calculated separately for the central region under the adherence, and for the lateral regions that remain under a locally mobile boundary. As adherence occurs between the background and the upper pole of the inclusion, the central portion under the adherence appears less stiff although the areas lying either side retain their stiff appearance caused by the local effects of a partial mobile boundary. The polar adherence that results in the divergence of the lines for the adhered and slippery zones corresponds with a drop in mean halo strain reflecting the loss of the mobile boundary.

Inclusions with a positive $C_{\mathrm{YM}}$ cause a strain concentration to appear above the inclusion as the soft background is strained against the stiff inclusion. Compression of the mobile boundary against the upper surface of the inclusion causes obliteration of the fluid layer at the upper pole of the inclusion as fluid is displaced and a point of adherence occurs between the background and the inclusion. The true strain image representation of $C_{\mathrm{YM}}$ is immediately revealed along a beam axial (vertical) line that includes this point of adherence where it behaves as an adhered inclusion but the mobile boundary regions over the lateral portions of the inclusion are not obliterated and the lateral zones of the inclusion beneath them retain their initial appearance. As the force increases, more fluid is displaced from the polar region and the zone of adherence spreads down the side of the inclusion toward the equator, accordingly the central zone of true strain representation of stiffness increases in size and the lateral zones decrease.

### 5.3 Isostiff and Soft Inclusions

**Fig. 12:**
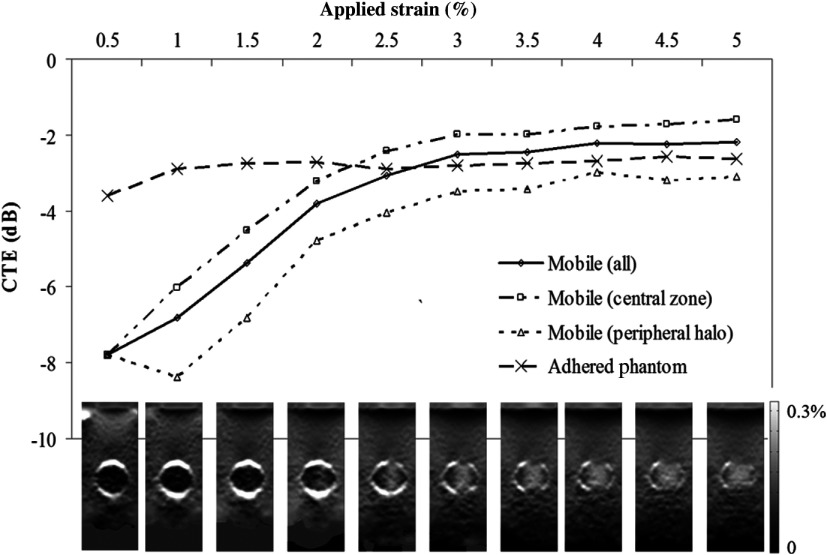
Zones of slip. CTE’ (signed) is plotted against applied axial strain for the slip and CTE (absolute) for adhered phantoms with a YMC of −4.27  dB (soft inclusion). For the slip phantom, signed CTE’ was calculated as Co—|Ct|. Four ROIs were measured: All = the entire inclusion. Slip (central zone) = central portion with a 20 pixel stand-off between the slip halo (image width=250  pixels). Slip (peripheral halo) = band (10 pixels wide) immediately inside the slip halo. Slip halo strain = mean strain in the slip halo. CTE (absolute) for the adhered phantom is also plotted. Strain is linear between 0% and 0.3%. Unscaled image is 25  mm×65  mm.

In order to demonstrate this phenomenon for a soft inclusion where contrast reversal occurs, Fig. 12 plots CTE that has been calculated using the signed rather than the absolute value of $C_{\epsilon }$ which we have termed CTE’ thus

$${\mathrm{CTE}}^{\prime }(\mathrm{dB})=C_{\epsilon }(\mathrm{dB})-|C_{\mathrm{YM}}(\mathrm{dB})|.$$

This results in a smooth transition from negative to positive contrast as CTE’ crosses the $C_{\mathrm{YM}}$ (−4.27  dB) in Fig. 12 rather than a dip toward it (as in Fig. 9), and more clearly illustrates the difference between the central and the peripheral ROI. CTE’ within the inclusion has been segmented into the peripheral zone (adjacent to the slip plane) and the central zone. The CTE’s for all ROIs are initially equal but the central zone ROI immediately rises above the global measurement to reflect a soft appearance. The peripheral zone rises at a slower rate and lies below the global inclusion measurement reflecting an annular “mantel” that appears stiff adjacent to the circumferential slip plane. The central zone also rises above the value for the adhered phantom demonstrating the softening effect compensating the peripheral low strain as strain is conserved within the inclusion. This explains the target appearance of mobile soft and isostiff inclusions on axial strain images: high strain in the center is related to true but amplified apparent softness, a circumference of low strain adjacent to the slip plane, and a rim or halo of high strain (the mobile boundary) and explains why mobile inclusions with no $C_{\mathrm{YM}}$ appear soft. This also provides experimental verification of the effect observed in the FEM simulations where altering the ROI radius results in differing strain values.

A possible explanation for the difference between inclusions with a positive $C_{\mathrm{YM}}$ and inclusions that have no $C_{\mathrm{YM}}$ or a negative $C_{\mathrm{YM}}$ is that it is not possible to force a stiff background against a soft inclusion or an inclusion with zero $C_{\mathrm{YM}}$, so the strain concentrations and polar adherence seen with stiff inclusions do not occur. Instead, there is uniform pressure distribution across the mobile boundary. Unlike the situation seen in stiff inclusions where polar adherence permits strain “escape” through the central subpolar region of the inclusion boundary, this does not occur with a continuous circumferential fluid layer and so the inclusion strains uniformly. As an additional effect, the mobile fluid layer causes low strain just inside the circumference of the inclusion, and because strain within the inclusion must be conserved, the center appears soft as it strains more to compensate the peripheral low strain. This is seen, albeit with elastographic noise, in the thumbnail images and the zonal analysis results of Fig. 12, and agrees with the FEM predictions in this paper.

## 6 Discussion

Figure 2(b) shows why the halo of high strain across the mobile inclusion boundary, as seen in Ref. 16 occurs. It is simply due to the discontinuous variation, and hence high spatial gradient, of displacement across the slippery boundary; its width is determined by the window length of the gradient-based strain estimator used in elastography, and its magnitude in turn will also depend on this variable. The reason for this discontinuity is the ability of the background tissue to pass freely over the inclusion surface. Rigid body rotation of the inclusion can be ruled out as its cause since appropriate constraints were placed upon the model. This also offers an explanation for why the halo “artifact” is seen in Ref. 16 but not in Ref. 17 for the former, a gradient-based strain estimator was used, and an element-based strain estimator was used in the latter. When the inclusion is connected to its surroundings, no sharp discontinuities exist in the displacement data, and strain images calculated in the two ways are very similar. When simulating the strain distribution of mobile inclusions, a gradient-based estimator must be used to more realistically reflect the situation observed in elastograms. An element-based strain estimation system would not show the halo-effect but, as in this paper, it may be used to analyze strain distributions in the absence of the effects of this “pseudostrain.”

The changes in strain distributions, in particular inside the inclusion, may, alongside visual observation and perhaps measurement of the halo-effect, provide an alternative method of determining whether or not the lesion is mobile, alongside dynamically monitoring motion in the B-mode images and axial shear strain assessment. The reason for the heterogeneous strain distribution in a mobile inclusion is not yet fully understood by us, although we can gain some insight by comparing the axial stress fields of the slippery and adhered inclusion models (Appendix C). The axial stress distribution in the adhered inclusion is homogeneous and heterogeneous in the mobile inclusion. From this result, we suggest that the internal variation in the mobile inclusion axial strain distribution is due to the heterogeneous transmission of stress into the inclusion due to the freedom of the background tissue to slip across the inclusion surface. In the adhered case, stress may penetrate into the inclusion to an approximately even extent across the inclusion boundary, resulting in homogeneous deformation of the inclusion. Further research is required to fully understand the differences in the internal stress and strain field distributions between mobile and adhered inclusions.

The reduction in mean strain in the mobile inclusion means that based on traditional elastographic interpretation, a mobile inclusion may appear stiffer on an elastogram than an adhered inclusion, independent of the Young’s modulus contrast and applied strain. This may lead to inaccurate interpretation of the elastogram without knowledge that it appears more stiff simply due to its mobility, rather than due to high Young’s modulus. We have shown that Young’s modulus contrast is not required to generate strain contrast between the inclusion and background, which is confirmed in Fig. 5(e). The strain distribution has a similar appearance to the stiff mobile inclusion in Fig. 2(f). Note that the nonzero CTE of the mobile inclusion at modulus contrast=0  dB is not due to the occurrence of the halo of pseudostrain, which has been excluded due to the method of CTE calculation. CTE is poorer for soft mobile inclusions than for soft adhered inclusions. For stiff mobile inclusions, CTE values gradually tend toward 0 dB with increasing modulus contrast. However, this does not mean that the elastograms are visually more similar to underlying Young’s modulus distribution, as is the case for an adhered inclusion of Young’s modulus contrast of 0 dB.

The mean strain in the halo of pseudostrain across the mobile inclusion boundary (Fig. 7) always has a higher value than the strain at the adhered inclusion boundary, for increasing applied strains and for all tested modulus contrast values. This is important if the examination of strain across tumor boundaries is to become a marker for mobility.

The simulations conducted here made several simplifying assumptions about the behavior of tissue, which future research should endeavor to use more realistic models to ensure that the predicted effects of mobility are as close to what may be seen clinically as possible. This includes the effects of both static and dynamic friction, fluid lubrication, irregular tumor shapes, heterogeneous background media, and 3-D modeling. Some of these physical phenomena are not simulated in the FEM study but are addressed in the experimental study. Furthermore, future research should also study the effects of mobility upon ultrasound echo data and the impact upon speckle tracking algorithms. An attempt to exploit decorrelation effects across mobile tumor boundaries for mobility assessment has been proposed by Kadour et al.,^62^ although insufficient information is provided in the publication to determine precisely how this is achieved. Finally, there should be assessment of the features associated with mobility noted here (high strain across the inclusion boundary, and characteristic strain heterogeneity within the inclusion and adjacent background medium) in terms of their diagnostic potential to discriminate between benign and malignant tumors. These features have already begun to be assessed in the brain^63^ for use in guiding tumor resection procedure.

The experiments reported here were performed to validate results from computer simulations of phantoms with mobile and adhered inclusions. With selection of frames from the experimental dynamic strain sequences, based upon an understanding of the limitations of simulations to accurately reflect the phantoms (which we believe to, in turn, more closely reflect the clinical situation), our analysis of the experimental data produced CTE results that agree with simulation in the following ways. Referring to Fig. 10 (which may be compared with the simulation results in Fig. 4), for negative $C_{\mathrm{YM}}$, CTE for mobile inclusions is below that of adhered inclusions at large negative modulus contrasts (here, −1.58 and greater). The implication is then that there is a small negative Young’s modulus contrast range (here, between data points at $C_{\mathrm{YM}}=-1.58$ and $C_{\mathrm{YM}}=0$) where the CTE of mobile inclusions is above that of adhered inclusions, rising above zero for a small range of very small negative contrasts. The CTE of mobile inclusions is greater than zero at a $C_{\mathrm{YM}}$ of zero and remains above the CTE for adhered inclusions for all positive $C_{\mathrm{YM}}$. The rate of change of CTE with inclusion modulus contrast, of both mobile and adhered inclusions, is less for stiff inclusions compared to soft inclusions, showing that for both mobile and adhered inclusions, a soft background transmits the applied strain into a hard lesion more efficiently than a hard background transmits strain into a soft lesion. All of these features are noted in the simulation results of Fig. 4 to facilitate comparison with Fig. 10. Stated briefly, a mobile boundary introduces a new strain contrast mechanism that is in addition to that due to any existing YM contrast, and this additional strain contrast is always negative when averaged over the whole lesion; an inclusion that is stiffer than its background will appear to have increased strain contrast, an inclusion that is much softer than the background will appear to have decreased strain contrast, an inclusion that is only slightly softer than the background may appear to have its strain contrast reversed so that it appears to be stiffer than the background, and an inclusion with no YM contrast will appear stiffer than the background.

### 6.1 Relationship to Modeling

An important difference between experiment and simulation was that the simulation did not observe several of the applied strain-dependent phenomena that were observed here experimentally, viz., variation in halo strain, variation in strain contrast, and contrast reversal. In simulation, a mobile inclusion boundary is achieved simply by setting the friction coefficient to zero. Experimentally, a lubricant is required. We, therefore, hypothesize that the additional phenomena observed experimentally were due to the use of fluid lubrication to achieve a mobile inclusion boundary. Specifically, fluid could be squeezed out of the space between the inclusion and the background, escaping from the phantom at the ends of the cylindrical rod-shaped inclusion. As applied strain increased, more and more fluid may have been squeezed out until points of adherent (i.e., unlubricated) contact between background and inclusion were created, generating the observed elastogram appearances of both local reduction in strain halo contrast and local increase in inclusion strain along the axial lines where this occurred. Eventually, at sufficiently high applied strain, most of the lubricant had gone and almost complete adherence existed around the inclusion boundary, producing almost complete loss of the strain halo and an adherent-type CTE result as seen in the adherent line in Fig. 10 (derived from the mean result from the adhered phantoms in Figs. 11 and 12, for inclusions that were designed to be mobile). Indeed, when small inclusion ROIs were used to measure strain contrast for only axial lines that included points of lost halo strain (at intermediate applied strain), this too resulted in an adherent-type CTE curve (Fig. 11), which seems to confirm that localized loss of halo strain corresponds to localized adherence. The one exception to this is the presence of the proximal halo strain seen in Figs. 11 and 12 at very small applied strains. We propose that this is due to the existence of so much lubricating fluid (i.e., an excess, relative to the amount necessary simply to achieve a mobile boundary) that the applied strain in these early compression steps is largely employed (in effect, absorbed) in producing a reduction in fluid volume, resulting in less strain than would otherwise be transmitted into the inclusion and distal background. This could therefore also account for the additional negative strain contrast and the contrast reversal for the soft inclusion phantom in these early compression steps, and the “hypermobile” CTE versus YM contrast curves seen in Figs. 8 and 9.

The behavior discussed in the last paragraph above is very different, in fact virtually the opposite, to that described for the mobile boundary elastography study of Chakraborty et al.,^64^ where an inclined plane phantom was used, and the applied force needed to produce slip (as judged by various elastographic criteria) was compared with the angle of the inclined plane and shown to agree with a simple model for the static coefficient of friction. However, Chakraborty et al. used very little lubrication at the inclined plane between two blocks of gelatine, which were thus designed not to slip until sufficient force was applied. In the present study, sufficient lubricant was initially present for slip to occur until the lubricant was squeezed out and adherent contact was made. It remains to be seen which model has the greatest clinical relevance and under which circumstances.

If the excess fluid hypothesis described above does indeed provide the correct explanation for the strain image behavior discussed above for the initial compression steps, then it is to be expected that the elastograms for these steps should exhibit a time-dependent behavior due to the finite time that it takes the fluid to flow and either redistribute around the boundary or escape at the ends of the phantom. In other words, the strain images should exhibit a poroelastic response^22^^,^^24^ at the instant immediately after a step in applied strain, before the lubricating fluid has had a chance to flow, the incompressible fluid should transmit the strain but lubricate the boundary, producing a strain image that may be comparable to that predicted by simulating a mobile inclusion. Subsequent fluid flow will cause strain relaxations to occur, which may result in the “hypermobile” elastographic behavior mentioned above. Future work could include a high time resolution strain imaging experiment to evaluate this conjecture. Meanwhile, the force relaxation observed and mentioned in the methods section above provides circumstantial evidence in support of this suggestion.

### 6.2 Clinical Significance

Physiological slip occurs in many anatomical locations in living organisms, where mobility is paramount to function. Examples include joints, tendons, and mobile internal organs such as the heart, lungs, and all abdominal viscera. In all these examples, motion is facilitated by a thin layer of lubricating fluid. Benign breast tumors, such as fibroadenomas, demonstrate slip behavior,^54^ which may or may not also be in the presence of a lubricating layer.

The fluid lubricated mobile boundaries in our phantoms mimic these physiological situations, and it is important to discuss the effects that this may have on clinical imaging in situations where fluid lubrication is present. We have shown that, even without fluid lubrication, a mobile boundary can cause a soft lesion to have the appearance of a stiff lesion and to alter the axial strain contrast and heterogeneity of both soft and stiff lesions. Our results also show, however, practical ways in which misinterpretation may be avoided. As suggested in the simulation data, a strong strain band (halo) at the boundary and the characteristic strain heterogeneity, where strain inside the lesion is reduced close to the boundary and increases toward the lesion’s center, if present, would indicate a slippery boundary. Here we have also shown that if fluid lubrication is present one may also be able to take advantage of the dynamic nature of quasistatic ultrasound elastography; initial strong strain halo and high negative strain contrast (lesion appearing stiff) at small applied strains, then, as applied strain increases, a decreasing strain halo and strain contrast, with possible contrast reversal if the lesion is softer than the background, would reveal its true characteristics. It is significant finding that for any lesion where these changes occur during compression, it can be inferred that it is mobile with a well-developed fluid lubricated slippy boundary. In the case of tumor boundaries, there is likely to be no invasion into the surrounding tissues (which may aid diagnosis) and in the case of intraoperative guidance it identifies a plane where atraumatic dissection may take place.

Predicting the location of atraumatic dissection planes is important during brain tumor surgery.^41^^,^^65^^,^^66^ Meningiomas, for example, are tumors that arise from the membranes surrounding the brain and grow into it. There is a physiological slip plane between the brain and its membranes (the subarachnoid space). As the tumor enlarges, it presses into the brain and although the subarachnoid space initially remains intact further growth results in loss of the subarachnoid space. Blood vessels on the surface of the brain may become surrounded by the tumor and eventually supply it through generation of new blood vessels. Eventually, direct brain invasion may occur. If the subarachnoid space is intact, a clear dissection plane exists throughout and these tumors are easily removed with little or no damage to the underlying brain. If the subarachnoid space has been breached, resulting in local areas of brain invasion, this rarely occurs over the entire brain–tumor interface. It is thus always easier to start the dissection in a region where a plane exists and work into an area where no plane exists, so location of a small area of slip is an advantage.

In summary, fluid lubricated mobile boundaries can be distinguished from adherent boundaries using the following criteria:

• Decrease in intensity of the strain halo as applied strain is increased.
• Increase in internal tumor strain as applied strain is increased.
• Decrease in internal tumor strain heterogeneity as applied strain is increased.
• Tumor strain contrast reversal, with increasing applied strain.

Evidence of these phenomena has been found in clinical scans (Uff, 2011) and this will be the subject of a future paper.

### 6.3 Alternative Mobility Indices

Other authors have proposed shear strain as a means of identifying whether a tumor boundary is mobile.^17^^,^^53^^,^^54^ Chakraborty^53^ used shear strain as a measure of when slip occurred as the force progressively increased and Coutts et al.^19^ showed the value of shear strain in detecting the fat-muscle slip plane. We have not evaluated shear strain in the present study; however, this may provide additional information in future work.

## 7 Conclusion

Whether-or-not an inclusion is mobile can dramatically affect the characteristics of axial strain elastograms. A halo of high strain occurs across the mobile inclusion boundary. The high spatial gradient of displacement at the slippery boundary, caused by the tissue background slipping past the inclusion surface, is sufficient to cause the strain halo, and it is not necessary to invoke tissue shear as an explanation, as was previously thought, although shear is also present. This halo could be used to provide additional diagnostic information. The presence of a slippery boundary lowers the mean strain in the inclusion, despite high strain concentration toward the inclusion center. Under traditional elastological interpretation, this creates a “stiffening” effect. If this is not accounted for, there is potential for misdiagnosis when interpreting strain in terms of stiffness. We have also shown that a mobile inclusion may have the same stiffness as its surroundings, but can still generate strain contrast. This calls into question the interpretation of elastograms in terms of stiffness, when signal can be generated due to effects other than Young’s modulus contrast. Mobile inclusions were found to exhibit an overall increase in strain distribution heterogeneity, which may therefore have potential as a discriminatory feature between benign and malignant tumors. An awareness of the results presented here should strengthen the diagnostic accuracy of axial strain elastography, enhance understanding of elastograms formed in the presence of slippery boundaries, and aid the generation of new image analysis methods for diagnosis and surgical guidance.

We have conducted experiments using phantoms intended to represent adhered and mobile tumors of varying Young’s modulus contrast to attempt to verify results seen in simulation. The absence of a mobile fluid layer in simulation led to important differences in the results, although when this was accounted for through rational selection of appropriate frames for analysis in the compression sequence, good agreement was obtained between experiment and simulation for CTE as a function of Young’s modulus contrast.

The presence of tumor mobility produces an apparent stiffening effect that when fluid lubrication is present, at particular levels of applied strain, can yield images of identical strain contrast, regardless of their true Young’s modulus contrast. Despite this effect, fluid lubricated mobile boundaries can be distinguished from adherent boundaries by varying the applied strain and using the following: a decrease in intensity of the strain halo, an increase in internal tumor strain, a decrease in internal tumor strain heterogeneity, and a possible tumor strain contrast reversal, with increasing applied strain. When palpation with the ultrasound probe is employed to create freehand quasistatic ultrasound strain elastograms, this may occur naturally during the examination. The results generated in this study will help guide interpretation of these images, which may help guide diagnosis and even surgical process.

## Figures and Tables

**Fig. 13:**
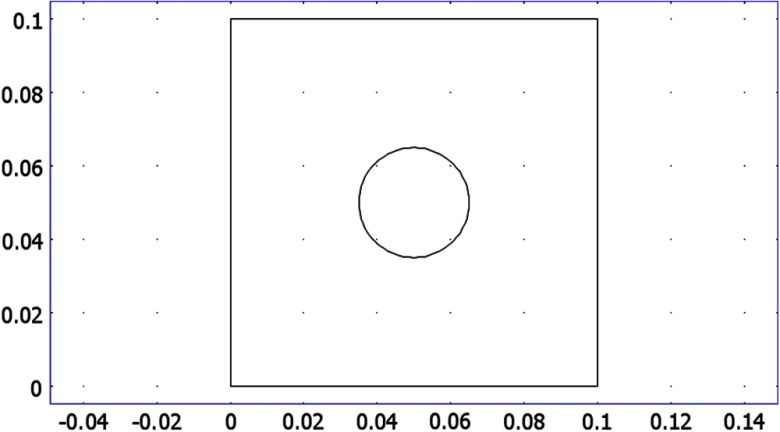
Illustration of the FEM used in all simulations.

**Fig. 14:**
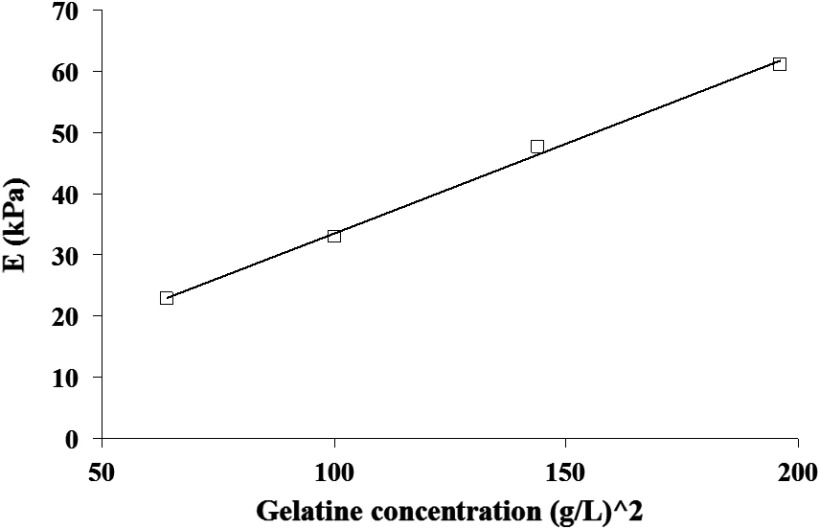
Least squared deviation best-fit straight line.

**Fig. 15:**
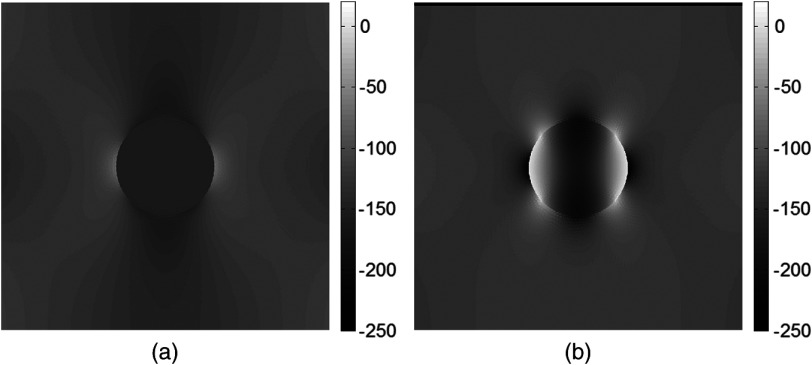
Illustration of axial stress fields resulting from 1% applied strain in (a) adhered and (b) mobile FEM simulation.

## Appendix A

Illustration of the FEM used in all simulations, with scale in meters. It is a 10-cm square with a 1.5-cm diameter inclusion. Image is exported from COMSOL (Fig. 13).

## Appendix B

Young’s modulus of gelatine samples, measured from force-displacement data obtained by controlled application of compressive strain along the long axis of the cylinders,^57^ as a function of gelatine concentration (by weight) squared. The least squared deviation best-fit straight line is also shown. The intersample variation of measurements was not explicitly investigated since only one sample was available from each batch of gel at a given concentration. However, the root mean-squared deviation of measured values about the linear least-squares best fit to YM versus the square of the gelatine concentration provides an indication that the intersample standard deviation is about 0.84 kPa, which at worst is no more than 4% of the mean YM (Fig. 14).

## Appendix C

Illustration of axial stress fields resulting from 1% applied strain in (a) adhered and (b) mobile FEM simulation. Inclusion modulus was 20 kPa, background 10 kPa, units in Pa. The axial stress distribution in the adhered inclusion is homogeneous and heterogeneous in the mobile inclusion (Fig. 15).
